# QUARITE (quality of care, risk management and technology in obstetrics): a cluster-randomized trial of a multifaceted intervention to improve emergency obstetric care in Senegal and Mali

**DOI:** 10.1186/1745-6215-10-85

**Published:** 2009-09-18

**Authors:** Alexandre Dumont, Pierre Fournier, William Fraser, Slim Haddad, Mamadou Traore, Idrissa Diop, Mouhamadou Gueye, Alioune Gaye, François Couturier, Jean-Charles Pasquier, François Beaudoin, André Lalonde, Marie Hatem, Michal Abrahamowicz

**Affiliations:** 1 Department of Obstetrics and Gynecology, Université de Montréal, Canada; 2 Department of Social and Preventive Medicine, Université de Montréal, Canada; 3 Research Centre of CHU Sainte-Justine, Université de Montréal, Canada; 4 CRCHUM Research Centre, Canada; 5 Centre de santé de la Commune V [Health centre, Commune V], Bamako, Mali; 6 Cabinet d'étude spécialisé dans la santé et l'action sociale (HYGEA) [Office for Specialized Studies in Health and Social Action], Dakar, Senegal; 7 Centre d'appui à la recherche et à la formation (CAREF) [Centre for the Support of Research and Training], Bamako, Mali; 8 Centre de santé [Health centre] Guédiawaye District, Senegal; 9 Department of Family Medicine, Université de Sherbrooke, Canada; 10 Department of Obstetrics and Gynecology, Université de Sherbrooke, Canada; 11 The Society of Obstetricians and Gynaecologists of Canada, Ottawa, Canada; 12 Department of Epidemiology and Biostatistics, McGill University, Canada; 13 Institut de recherche pour le développement, UR 010, Dakar, Sénégal

## Abstract

**Background:**

Maternal and perinatal mortality are major problems for which progress in sub-Saharan Africa has been inadequate, even though childbirth services are available, even in the poorest countries. Reducing them is the aim of two of the main Millennium Development Goals. Many initiatives have been undertaken to remedy this situation, such as the Advances in Labour and Risk Management (ALARM) International Program, whose purpose is to improve the quality of obstetric services in low-income countries. However, few interventions have been evaluated, in this context, using rigorous methods for analyzing effectiveness in terms of health outcomes. The objective of this trial is to evaluate the effectiveness of the ALARM International Program (AIP) in reducing maternal mortality in referral hospitals in Senegal and Mali. Secondary goals include evaluation of the relationships between effectiveness and resource availability, service organization, medical practices, and satisfaction among health personnel.

**Methods/Design:**

This is an international, multi-centre, controlled cluster-randomized trial of a complex intervention. The intervention is based on the concept of evidence-based practice and on a combination of two approaches aimed at improving the performance of health personnel: 1) Educational outreach visits; and 2) the implementation of facility-based maternal death reviews.

The unit of intervention is the public health facility equipped with a functional operating room. On the basis of consent provided by hospital authorities, 46 centres out of 49 eligible were selected in Mali and Senegal. Using randomization stratified by country and by level of care, 23 centres will be allocated to the intervention group and 23 to the control group. The intervention will last two years. It will be preceded by a pre-intervention one-year period for baseline data collection. A continuous clinical data collection system has been set up in all participating centres. This, along with the inventory of resources and the satisfaction surveys administered to the health personnel, will allow us to measure results before, during, and after the intervention. The overall rate of maternal mortality measured in hospitals during the post-intervention period (Year 4) is the primary outcome. The evaluation will also include cost-effectiveness.

**Trial Registration:**

The QUARITE trial is registered on the Current Controlled Trials website under the number ISRCTN46950658 .

## Background

### Maternal and perinatal mortality in sub-Saharan Africa

In sub-Saharan Africa, maternal and perinatal mortality and morbidity are major problems for which progress has been inadequate. Reducing them is the aim of two of the Millennium Development Goals (MDG4 and MDG5) whose attainment in this region of the world is very unlikely [1]. The broad strategies that have made it possible to reduce maternal and perinatal mortality are known: prenatal care, labour and delivery management by qualified personnel, and availability of emergency obstetric care (EmOC) [2]; however, their implementation is a major challenge in sub-Saharan Africa, where healthcare systems are fragile and still being developed. Service availability and quality of care in health facilities are very heterogeneous and most often inadequate [3-7]. According to WHO, it will be important, over the next 10 years, to update the skills of many professionals who do not currently have the competencies required to provide EmOC [8]. In addition, among the trained professionals currently on staff, it is apparently difficult to maintain a high level of performance in services that are often disorganized and under-equipped [9]. The lack of motivation leads to a high level of staff turnover, with responsibilities then falling on categories of staff that are less qualified [10].

In Mali and Senegal, the rates of maternal mortality estimated by WHO in 2005 remain high: 970 and 980 maternal deaths per 100,000 live births, respectively [8]. Emergency obstetric care coverage is poor (around 15%) [5,7]. On the other hand, according to UN indicators, there are enough referral centres equipped with functional operating rooms. However, the quality of care in the referral centres is inadequate, as evidenced by high case fatality rates (above 1%) [5,7].

### Interventions to improve the performance of health professionals

The concept and techniques of continuous quality improvement offer a variety of strategies to improve the performance of health professionals [11]. These approaches relate to complex interventions in which health professionals are directly involved in analyzing and modifying care processes to improve their performance and the health outcomes of their patients.

A meta-analysis of educational outreach visits, including 69 randomized controlled trials, most of which were carried out in industrialized countries, shows moderate effects in terms of changing professional practices, with considerable heterogeneity depending on professional categories and contexts [12].

A meta-analysis of audit and feedback approaches that reviewed 47 randomized controlled trials with more than 3,500 clinicians shows that this technique may be effective in improving medical practices. The baseline compliance with recommended practice (prior to intervention) and the intensity of audit and feedback are major factors influencing the effectiveness of this technique [13].

In low-income countries, a systematic review of interventions aimed at improving the performance of health professionals suggests that: (i) simple dissemination of written guidelines is often ineffective, (ii) supervision and audit with feedback are generally effective, and (iii) multifaceted interventions might be more effective than single interventions [9].

The ALARM (Advances in Labour and Risk Management) International Program, or AIP, was developed by the Society of Obstetricians and Gynaecologists of Canada (SOGC). It is based on a combination of two potentially effective approaches for improving performance among health professionals [14]: 1) educational clinically-oriented and evidence-based outreach visits focused on the principal causes of maternal mortality, and 2) facility-based maternal death reviews or audits, as proposed by WHO [15]. In 2005, we conducted a pilot study in Senegal to analyze the feasibility of maternal death reviews in five referral hospitals [16]. The results of this study confirm that the majority (87%) of maternal deaths in referral hospitals are attributable to direct obstetric causes, and three-quarters of these can be avoided using locally-adapted measures. The results also suggest that the involvement and leadership of those in charge of maternity services (physician and midwife) play a primordial role in implementing audits. The capacity of those in charge of the service to ensure such leadership depends on the following key aptitudes: (i) knowledge of evidence-based practice for the main obstetric complications; (ii) an understanding of non-medical reasons for maternal death (social, economic, cultural, and legal dimensions of maternal mortality); and (iii) a mastery of the clinical audit approach. We also showed, in another study carried out in a district hospital in Senegal, that the routine conduct of maternal death audits led to a reduction in maternal mortality of 50% in year 3, in comparison with the pre-intervention period [17].

### What is not known

While the results of some observational studies carried out in sub-Saharan Africa are promising enough [17-19], we have no evidence regarding the effectiveness, in terms of reducing maternal and perinatal mortality, of interventions based on educational outreach visits and audits, nor on their large-scale implementation. For reasons of cost and availability of information, studies on maternal and perinatal health interventions in developing countries are based preferentially on process indicators rather than health outcomes indicators [3-7].

From our pilot study in Senegal, the following questions emerged: 1) Does the AIP promote the development of local leadership? 2) Does the AIP produce changes in clinical practice among the health professionals? 3) Do the changes in practice have an effect, in the medium term, on maternal and perinatal mortality? 4) Does the AIP have an effect on staff satisfaction? 5) Can the intervention model be modified to improve cost-effectiveness? The QUARITE trial is an attempt to respond to these questions and to the need for more evidence on the effectiveness and functioning of interventions aimed at reducing maternal and perinatal mortality and morbidity in developing countries.

### Hypotheses

Our hypothesis is that the ALARM International Program (AIP) reduces the overall rate of maternal mortality, as measured in the hospitals in the post-intervention period, by 30% in comparison with the control group. Secondary hypotheses are that the AIP: 1) reduces the number of stillbirths and early neonatal mortality; 2) reduces severe maternal morbidity and the case fatality rate; 3) improves the quality of care through better utilization of local resources and changes in professional practices; and 4) increases the satisfaction of health professionals.

### Aims

The objective is to evaluate the effectiveness of the AIP in reducing maternal mortality at referral hospitals in Mali and Senegal, as well as improvements in perinatal health, resource availability, service organization, medical practices, and the satisfaction of health personnel.

### Design

This is an international, multi-centre, controlled cluster-randomized trial of a complex intervention. To avoid contamination bias between clinicians in the same service, the unit of randomization and of intervention is the participating healthcare facility.

### Inclusion and exclusion criteria

The hospitals entered the trial in September 2007. The study will be conducted in 46 out of a total of 49 eligible referral hospitals—23 in Mali and 26 in Senegal—spread across both countries. A hospital was eligible for the trial if it had functional operating rooms and carried out more than 800 deliveries annually. Three eligible hospitals were excluded: two already had a structured program for carrying out maternal death audits before the project began, and for one other hospital, written consent was not provided by local authorities. The 46 included hospitals are representative of the existing health system in Senegal and Mali, taking into account the variety of the contexts (urban versus rural) and of the levels of care (primary versus secondary referral health facilities).

The intervention directly targets health professionals involved in obstetric care in the various participating hospitals and, indirectly, the women who give birth in these facilities. These health professionals are the staff who are trained in labour and delivery management and the clinical management of obstetric or neonatal complications: physicians, midwives, obstetric nurses, nurse-anaesthetists, and surgical assistants.

Inclusion criteria for women in the QUARITE study are 1) being a patient who delivered in one of the participating facilities, 2) between September 1, 2007, and August 31, 2011. Exclusion criteria are 1) having delivered at home or 2) in another centre, with postnatal transfer.

### Intervention group activities

These activities started in September 2008 and will end in August 2010. Professionals from the intervention group have been trained in evidence-based practice using the ALARM international course [14] that targets health professionals who provide obstetrical care, reviewing the top maternal killers and suggesting essential tools and problem management with the goal of improving care for mothers and newborns. This course promotes evidence-based practice, using data from up-to-date systematic reviews of randomized trials. The course was developed and is maintained and taught jointly by family physicians, obstetricians, midwives, and nurses from developing countries. It has the administrative support and backing of the SOGC.

**Figure 1:**
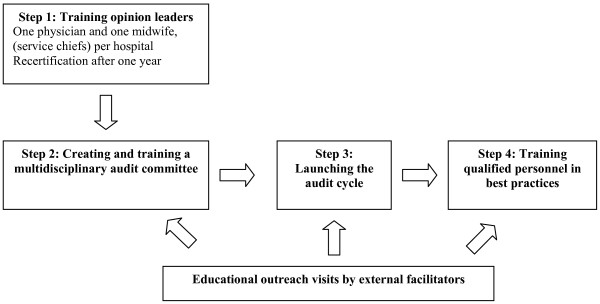
**Key steps in the implementation of the ALARM International Program in each hospital of the intervention group**.

The sequence of activities during the two years is directed toward developing local leadership and empowering obstetric teams. To meet this objective, the intervention will be carried out in several steps (Figure 1). The intervention began with recruitment of opinion leaders in each centre and their training in best practices and in maternal death audits (training the trainers). The opinion leaders will then create, in their own centres, obstetric teams charged with implementing the maternal death audits and will organize staff training in best practices with the support of external facilitators (educational outreach visits).

**Figure 2:**
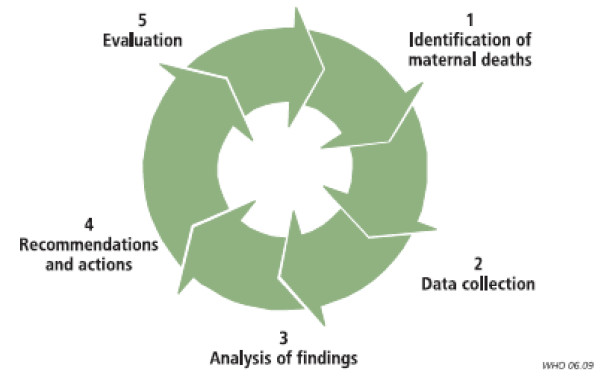
**Maternal death audit cycle in the hospitals**. Source: Dumont et al [17].

a) ***Training opinion leaders***: The local opinion leaders are those responsible for maternity services in the hospitals of the intervention group (one physician and one midwife per centre). In September 2008, the opinion leaders took part in a six-day training session provided by three certified instructors. The session included three days of training in EmOC best practices, one day of training in maternal death audit techniques, one day of awareness training related to economic, sociocultural and ethical barriers (including sexual and reproductive rights), and one day of training in adult education methods. At the end of the session, a normative evaluation was carried out. No financial inducement was given to the health workers involved in the program at the intervention sites.

b) ***Creating and training a multidisciplinary audit committee ***(physicians, midwives, nurses, and administrators), according to the following agenda: 1) identification and training of data collectors on maternal deaths; 2) training of committee members in the process of carrying out audits; and 3) annual summary of audit results.

c) ***Launching the audit cycle***: With the support of external facilitators, the audit process will be set in motion by the audit committee in each centre in accordance with the approach proposed by WHO [15] (see Figure 2). Monthly audit meetings are recommended to analyze cases of maternal deaths in the facility.

d)***Training qualified staff in best practices***: The local opinion leaders, supported by the external facilitators, will train the professionals in each health facility according to the following agenda: 1) evaluation of needs for best practices training; and 2) organization of between four and eight training sessions in best practices during the intervention period. The themes will be selected by local opinion leaders according to the principal causes of maternal mortality that will be identified during maternal death reviews.

d) ***Educational outreach visits by external facilitators***: A national opinion leader, chosen for his or her expertise and leadership, and the AIP international coordinator will visit all the hospitals in the intervention group every three months. The purpose of these visits will be to support the local opinion leaders in their role of providing AIP training to all the health professionals in the maternity service and to ensure that audits are being carried out, particularly by overseeing an audit meeting. To promote evidence-based practice, meetings with the hospital's professionals and administrators will be encouraged, as well as clinical observation periods related to the themes that were taught.

e) ***Recertification of the opinion leaders***: One year after the initial training (September 2009), the opinion leaders of both countries will undergo an accelerated training session together, led by certified instructors. The purpose of this recertification session is to verify the opinion leaders' knowledge, update them on the clinical content and process of maternal death audits, discuss their roles, share their experiences and confirm their capacity to provide leadership in their clinical settings.

### Control group

No external intervention is planned for this group.

### Study endpoints

Endpoints will be measured including periods before (12 months), during (24 months), and after the intervention (12 months). The main reason for the 12 month pre-intervention data collection was to measure process and outcome indicators during the baseline period and to control the effect of the intervention for possible changes in these indicators within the four-year study period.

The primary endpoint measure is the overall rate of maternal mortality (number of maternal deaths among women giving birth in the facility). The sample size was estimated in order to ensure adequate statistical power to show significant differences in maternal mortality among the groups.

As secondary endpoints, we also defined three levels of measurement for effectiveness of the intervention:

***a) Indicators of resource availability***: A systematic and standardized inventory of available resources will be undertaken each year. The hospital complexity index will be calculated for each facility to reflect the availability of different categories of resources required to provide high quality emergency obstetric care [20]: basic services, screening tests, basic emergency obstetric resources, intrapartum care, general medical services, anaesthesiology resources, human resources, academic resources, and clinical protocols.

***b) Indicators of quality of care***: The clinical data collected will allow us to measure the rates of essential obstetric interventions considered effective in reducing maternal and perinatal mortality: assisted deliveries (forceps and vacuum extraction), caesarean sections, transfusions and hysterectomies, transfers to other heath facilities. The quality of care will also be evaluated on a sampling of cases according to objective criteria for clinical management (criterion-based clinical audit). Repeated satisfaction surveys of the health personnel will allow us to measure, on the one hand, the staff retention rate, and on the other, changes in the level of satisfaction among professionals. Staff movements will be monitored as part of the health workers satisfaction study, for which we undertake an annual census.

***c) Maternal and perinatal health outcomes***: The incidence of maternal mortality will be measured, as well as its distribution among its principal causes: pre- and postpartum hemorrhage, prolonged or dystocic labour, uterine rupture, postpartum infection, pre-eclampsia/eclampsia. We will also measure, in each facility, the case fatality rate, the rate of stillbirths, and early neonatal mortality (during hospitalization). We will monitor referrals in and out of the facilities and track the mortality of mothers and babies discharged from the institutions, using the existing information systems (hospital registers and feedback from referral institutions to the others) and calling the institutions when the information is not available.

**Figure 3:**
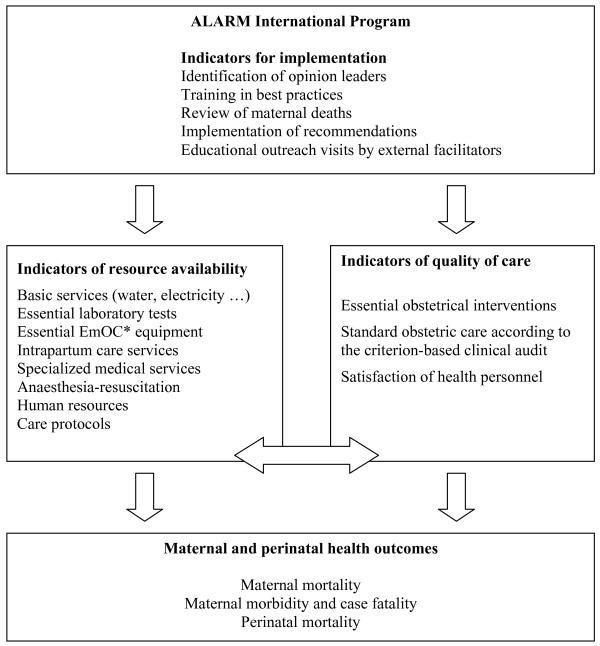
**Outcomes of the trial**. *EmOC: Emergency obstetric care.

Audit of maternal deaths is the cornerstone of this complex intervention. According to WHO [15], audit of maternal deaths should improve inter-personnel communication, team working (and staff motivation), resource availability, performance of health workers, and quality of care. Training for the local opinion leaders and parallel training for other staff in best practices should reinforce the effects of the audit activities on health workers' performance. The other components of the intervention should facilitate the implementation of the program using activities to support the actors (outreach visits). As with other complex interventions, the synergy among all components is expected to be effective. All these effects will ultimately have an impact on maternal mortality (see Figure 3). The effects of each component will be assessed by measuring the process indicators: indicators for the implementation of the program, and indicators of resource availability and of quality of care.

### Randomization and allocation

Centres were included on the basis of formal, informed consent on the part of the hospital director and the person in charge of maternity services. After a one-year pre-intervention data collection phase, each hospital was randomly assigned, in August 2008, to either an intervention group, in which the AIP will be implemented, or a control group.

**Figure 4:**
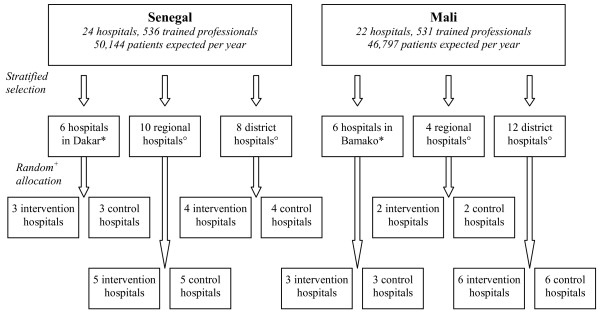
**Trial design**. *The hospitals in each country's capital (Dakar in Senegal and Bamako in Mali) are characterized by a high level of obstetric activity (more than 3000 deliveries annually) and are easily accessible to the public because of their proximity. °Regional hospitals and district hospitals are outside the capitals. ^+^Randomization by blocks of 2 in order to ensure a comparable number of patients in both groups.

The participating hospitals were stratified in six strata corresponding to the combination of two countries (Mali and Senegal) and three hospital types: hospitals in the capital, regional hospitals, and district hospitals outside the capital. We attempted to ensure optimal balance between the hospitals assigned to the intervention and the control groups in terms of their number and size (number of deliveries per year). Therefore, within each stratum, we first ranked the hospitals with respect to size, and then used blocked randomization, with each block of size two containing two hospitals with adjacent ranks, i.e., of similar size (Figure 4). All participating hospitals were randomized simultaneously, after their list was provided, which eliminated any risk of allocation bias.

### Data collection and management

#### Data collection

A system of data collection, independent of the AIP, was set up in September 2007 in the 46 study hospitals. This system is based on the WHO global survey on maternal and perinatal health [20], which considers clinical data at the individual level and organizational data at the facility level. All deliveries carried out in the participating centres are registered by local collectors (nurses or midwives trained to do this). These collectors complete a standard form for each eligible patient that includes information on maternal characteristics, prenatal care, labour and delivery, diagnosed complications, and the vital status of both mother and child at discharge from hospital (see patient registration form, Additional file 1). This information is extracted from the hospital registers and from available medical records whose quality and archiving are regularly monitored by the national coordinator of the study. These data will be collected on an ongoing basis throughout the study. With respect to facility-level data, the national coordinator carries out an annual inventory of the resources available in each hospital using a standardized grid, by visiting all the services of the hospitals involved in EmOC.

To assess the quality of care, we will sample medical records of 50 patients at each of the 46 participating centres using simple random sampling. An external evaluator will assess each record using a criterion-based clinical audit (CBCA) to obtain a score for quality of care. The CBCA scores, ranging from 0 to 27, are then dichotomized as standard (score equal to or greater than 22) versus sub-standard (score below 22) care. The sample size of 50 records/centre will allow us to estimate the proportion of patients receiving standard care in each centre with sufficient precision. Specifically, the half-width of the 95% confidence interval will not exceed 15%. The CBCA developed by the researchers of the QUARITE trial employs evidence-based clinical criteria to ascertain if minimal quality of care is provided to the patients sampled. These criteria have been selected based on thorough review of the literature and expert consensus and are consistent with the WHO guidelines for conducting an obstetrical CBCA (Beyond the numbers: Reviewing maternal deaths and complications to make pregnancy safer. Geneva: World Health Organization, 2004) [15].

Before, during, and after the intervention period, satisfaction surveys will be conducted with all the health professionals in the participating centres; these will be done by a research professional in individual interviews. We will use a pre-tested questionnaire that contains 42 closed, Likert-type questions. The questions on satisfaction relate to remuneration, work tools and environment, work organization and content, training and supervision, emotional satisfaction, management style, and attitudes toward change. As part of these surveys, staff censuses are undertaken in all the health facilities included in the study.

### Data management

Patient records are collected by the national coordinator during quarterly visits and transferred to the national coordinating centre for double data entry using Epi-Info 2000 software. The electronic record, cleared of each quarter's clinical data, is then transmitted to the trial's coordinating centre at the University of Montreal and stored in a secure location. The facility-level data and the data regarding staff satisfaction are recorded separately. The different databases are periodically verified by the data manager. All information collected on patients, health professionals or facilities is confidential. Access to the clinical database is restricted to the data manager until the end of the study. Access to the other databases is restricted to the coordinators and researchers responsible for the various sections of the trial.

#### Quality control of data

The quality control of clinical data will be carried out in three stages. The first stage corresponds to the quarterly visits of the national coordinator. During these visits, the coordinator verifies that the data collection is exhaustive by comparing the number of eligible patients on the hospital's birth register with the number of patient forms collected. A complementary procedure is carried out to monitor the thoroughness of data on maternal deaths, which are generally under-registered in the maternity ward, by identifying the eligible maternal deaths among all the female deaths that occurred in the facility using the various registries available: admissions, hospitalizations (maternity and other services), operating rooms, and morgue. On a random sample of patient forms, the coordinator checks the quality of data collected. The completion rate will be estimated as the proportion of patient forms that contain 100% of the following information: date of entry, patient identification, date of discharge, and vital status of the mother at the date of discharge. The concordance rate will be estimated as the proportion of patient forms whose information is concordant with the hospital registers and medical records. Both the completion rate and the concordance rate are expected to be above 75%. If the completion or concordance rate is between 50% and 75%, the coordinator will check the data quality on a new random sample of patient forms. A completion or concordance rate of less than 50% will trigger the verification of all patient forms. A second control of missing or abnormal data will be carried out at the national coordination centres before data entry. If necessary, the missing information is obtained from the collectors of each facility by telephone or fax. The third step is carried out by the data manager after the data has been entered and transmitted to the trial coordination centre. An audit report of the database, including lists of duplications and missing or abnormal data, is sent quarterly to the national coordination centres, which are responsible for correcting any errors.

### Statistical analysis plan

#### Sample size and power calculations

The formula for calculating the required number of patients is that used for a cluster-randomized controlled trial design [21]. The calculation is based on an overall maternal mortality rate of 1.5% in the pre-intervention phase and an expected reduction of 30% in maternal mortality in the hospitals of the intervention group, in comparison with the control group. This rate was estimated as an approximate average of stratum-specific rates, which ranged from 0.3% to 2.7%, observed in Senegal in a pilot study in 2004-2005 [16]. Taking into consideration a minimum of 830 women included per hospital, the number of patients required was calculated for an odds ratio of 0.70 and for an intra-cluster correlation coefficient (ρ) of 0.001. The value of 0.001 can be considered conservative given the results of the WHO global survey on maternal and perinatal health [22]. The calculation shows that a total of 38,205 patients and 46 hospitals (38,205/830) allows us to achieve a power of 82% to detect a 30% reduction in the overall rate of maternal mortality between the two groups (OR = 0.70) with 2-sided significance test at α = 0.05 and with ρ = 0.001 (ACluster-design^® ^2005, version 2.0, World Health Organization).

According to service statistics available for 2006 and collected for the 46 hospitals in the study, the expected number of deliveries is 96,941 (mean cluster size = 2300; minimum = 830; maximum = 8600), 50,144 in Senegal and 46,797 in Mali. We identified 1,067 trained professionals working in these facilities, 536 in Senegal and 531 in Mali.

#### Type of analysis and handling loss-to-follow-up

The acceptability of the various components of the AIP, including maternal death audits, is high [16,23]. Nevertheless, it is possible that a few hospitals may decide to withdraw from the study. For these hospitals, data collection will continue until the end of the study, in accordance with the commitment made by the hospital authorities at the time of inclusion. These hospitals will therefore not be excluded from the analysis. We will conduct intention-to-treat analyses: each patient will be analyzed in the hospital where she was admitted and each hospital will be analyzed within the group to which it was originally assigned by the randomization.

#### Statistical analyses

Binary outcome (maternal death) will be measured for each woman included in the study, while the unit of randomization and intervention is the hospital, to avoid contamination bias. A first descriptive analysis will allow us to verify, in the pre-intervention phase, the comparability of the groups in terms of the characteristics of the centres and of the patients included. The primary analyses will take into account the inter- and intra-cluster variability and adjust the intervention effect for stratification variables (country and hospital type). To adjust the estimate of variance in the intervention's effect on the interdependence of the events measured (maternal death or not) among the patients of a given hospital, we will use the Generalized Estimating Equations (GEE) approach which generalizes logistic regression to cluster data [24]. The interchangeable structure of the residual covariance matrix will be used to represent intra-cluster correlations. In secondary analyses, we will use the multivariable GEE model to adjust the effect of the intervention for those patient-level or hospital-level variables that show a marked imbalance between the two arms of the trial. The same approach will be used for secondary assessment criteria. In all the analyses, the effect of the intervention will be estimated using the odds ratio (95% CI) from the GEE model and tested by one-tailed Wald testing (α = 0.05) [24].

The preliminary analyses will verify whether the intervention's effect varies according to country using the likelihood ratio chi-square test with one degree of freedom for country-intervention interaction. If the test does not reject the null hypothesis at α = 0.05, the interaction will be eliminated from the final model, which will allow us to assess the overall effect of the intervention. On the other hand, if the interactions are statistically significant, the sub-group analyses will assess the effects of the intervention separately for each country, with a power less than 90%. A similar approach will be used to test whether the effect of the intervention depends: (i) on the overall rate of maternal mortality in the pre-intervention phase (Year 1); (ii) on changes in maternal mortality within the intervention period; or (iii) on hospital type.

#### Economic evaluation

The economic analysis has two components. First, an incremental analysis will be undertaken to evaluate the cost per life saved in each of the hospital strata. Second, a simulation will be conducted of the overall benefits and marginal costs of a hypothetical scaling up of the maternal audit intervention at the national level. Costs include both direct costs of the intervention (employees, consumables, equipment, overheads, and capital costs) and costs incurred by other units and patients, following changes in the practices of the newly trained professionals. Key parameters of the analysis, such as activities and resources mobilized for the intervention, real prices, attribution rules for capital costs and overheads, and unit costs in each hospital stratum, will be determined during a preliminary survey. The measurement of costs will be based on project cost-sheets, reports, budgets, and interviews. Multiway sensitivity analysis will be performed to assess robustness of the results.

### Potential limitations of the trial

Although there is no "intervention", the activity related to data collection may have an impact on quality improvement and maternal outcomes in the control group. However, this "data collection" effect is *a priori *similar in the two groups (intervention versus control). The results of our trial may be contaminated if hospital staff transfer from centres randomized to an active intervention group into other centres included in the control group. Such contamination would attenuate the apparent effectiveness of the intervention. To assess the extent of such potential contamination, we will keep track of all transfers of the hospital staff involved in the trial between the study centres. Up to now (end of Year 1), the rate of personnel transfers is low (less than 1%). Informal data obtained from interviews with the personnel during the coordinating visits suggest that most of the staff in the active-intervention centres do not want to be transferred before the end of the trial, which should limit the risk and the extent of such potential contamination. Furthermore, implementation of the AIP requires sustained teamwork unlikely to be assumed by isolated individuals.

### Ethical issues

#### Ethic committee approvals

The trial has been approved by the ethics committee of Sainte-Justine Hospital in Montreal, Canada, which manages the operating funds, and by the national ethics committees in Senegal and in Mali. The QUARITE trial is registered on the Current Controlled Trials website under the number ISRCTN46950658 .

#### Informed consent and information sheet

The participating hospitals were included on the basis of informed consent by the local authorities (director of the centre and chief of maternity services). The authorities were informed that 1) all centres had the option of withdrawing from the project at any time; 2) the AIP training would be offered to the control group hospitals at the end of the study if the intervention proved to be effective; and 3) data collection would continue until the end of the study, even for centres that withdraw from the study. Consent was also obtained from staff who participated in the satisfaction surveys. Collection of clinical data from hospital registers and medical records is authorized by the hospital authorities and does not require patient consent [20].

#### Interim analyses and stopping rules

An independent data security and monitoring committee (DSMC) was established, made up of three international experts in epidemiology and biostatistics, reproductive health research, and obstetrics and gynaecology in resource-poor settings. Its primary responsibility will be to ensure the security of the trial and to monitor the progress of the research according to the established protocol. The committee could decide to stop the trial for the following reasons: if the AIP is proven effective by the end of the first year of intervention; if the quality of the data is inadequate; if the level of implementation of the AIP among the hospitals in the intervention group is too low; if there is significant contamination of the control group; if there is fraud; if there is any new information that would lead to the conclusion that the trial is unnecessary, useless, or even unethical. To assess the effectiveness of the intervention at the end of the first year of intervention, an intermediate analysis will be planned using Peto criteria (α = 0.001), which ensure a total type I risk of error, for the final analysis, of 0.05. If, based on the results of this interim analysis, the DSMC decides to discontinue the trial, we will continue long-term follow-up to monitor the implementation of the program and the reduction of maternal mortality in the two groups (intervention and control).

### Sponsor and project administration

**Figure 5:**
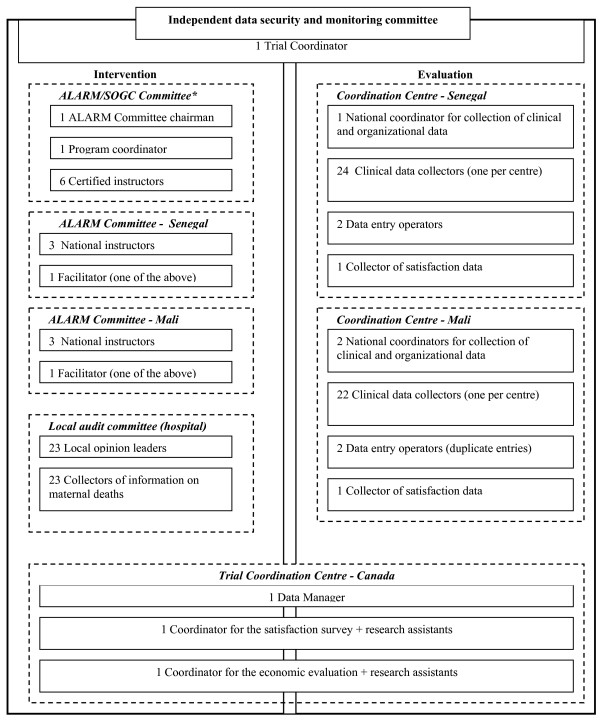
**Administrative structure for the trial**. *SOGC: Society of Obstetricians and Gynaecologists of Canada.

The project was approved by the Canadian Institutes of Health Research (CIHR) and funded in January 2007. The trial began in September 2007 in Senegal and in November 2007 in Mali. The funds are managed by Sainte-Justine Hospital of the University of Montreal. The multidisciplinary committee is made up of Canadian, Senegalese, and Malian researchers with expertise in public health, maternal and perinatal health, clinical research, medical teaching, and biostatistics, with a strong global health orientation. The collaborating centres in Senegal and Mali, specializing in large-scale data collection and entry, are also involved in the study. The ALARM International Program will be coordinated by the SOGC in collaboration with the Ministry of Health, the Faculty of Medicine and the professional associations (gynaecologists-obstetricians and midwives) of each country. The project's administrative structure is presented in Figure 5. The monitoring of the intervention and evaluation components is carried out by the trial coordinator, who reports regularly to the steering committee.

The trial steering committee meets twice a year to oversee the implementation of the ALARM International Program in the intervention group, follow the data collection process, monitor data quality, coordinate the intermediate statistical analyses, consider the conclusions of the independent committee on data security and monitoring, and coordinate the economic evaluation.

**Start date**: September 1, 2007

**Intervention period**: September 1, 2008 to August 31, 2010

**End date**: August 31, 2011

**Reporting date**: January 1, 2012

## Competing interests

The authors declare that they have no competing interests.

## Authors' contributions

AD participated in developing the project and is responsible for the scientific aspects of the trial and all its components. PF participated in developing the project and is responsible for its administration and for coordinating the satisfaction survey. MA participated in developing the project, will plan the analysis, and will coordinate the statistical analyses. WF participated in developing the project and ensures the trial is carried out in accordance with best practices. SH is responsible for the economic evaluation and rewrote this section in the revised paper. AG, MT, FB, FC, MH, JCP, and AL participated in developing the project and are responsible for the intervention. ID is the Director of the coordination centre in Senegal (HYGEA) and is responsible for the collection of clinical and organization data in Senegal. MG is the Scientific Director of the coordination center in Mali (CAREF) and is responsible for the collection of clinical and organization data in Mali. AD wrote the first version of the protocol and, with PF, coordinated its development and approved the final version. AD, PF, MA, WF, FB, FC, MH, and JCP obtained the funding for the project. All authors provided feedback and made revisions to the manuscript.

## Supplementary Material

Additional file 1 **Appendix 1**. Patient registration form. Click here for file
