# Supplementary material for: QUARITE (quality of care, risk management and technology in obstetrics): a cluster-randomized trial of a multifaceted intervention to improve emergency obstetric care in Senegal and Mali
Source: Trials. 2009 Sep 18;10:85. doi: 10.1186/1745-6215-10-85 (PMC2758868; doi:10.1186/1745-6215-10-85)
Supplement: Supplementary file 1 [file 1745-6215-10-85-S1.DOC]

Partie réservée à la saisie	Numéro de saisie :	Code Pays :	Code établissement :

**I.IDENTIFICATION (confidentiel)**	Nom de l'établissement:
Nom/prénom de la patiente :	
Adresse: (Région/Ville ou Département/Quartier ou village)	Numéro d'enregistrement:

II.CARACTERISTIQUES MATERNELLES

Q1.Age	/__/__/	Q2.Gestité	/__/__/	Q3.Parité	/__/__/	Q4.Antécédent de césarienne	☐ 1 Oui	☐ 0 Non

**III.GROSSESSE ACTUELLE**		Q5.Nombre de consultations prénatales (CPN)	/__/__/
Q6.Pathologies diagnostiquées *si oui aller à Q7 ; si non aller è Q8*	☐ 1Oui	☐ 0 Non	Q7.Préciser:
Q8.Type d'admission:	☐ 1 venue d'elle même	☐ 2 évacuée	Q9.Date d'admission: (jour / mois / année)

**IV.TRAVAIL ET ACCOUCHEMENT**	Q10.Patiente décédée avant le travail *si oui aller à Q14, si non aller à Q11*	☐ 1 Oui	☐ 0 Non
Q11.Début de travail	☐ 1 spontané	☐ 2 déclenchement / induction	☐ 3 pas de travail
Q12.Mode d'accouchement	☐ 1 vaginal simple	☐ 2 Forceps/ventouse	☐ 3 siège (assisté)
☐ 4 césarienne en urgence avant travail	☐ 5 césarienne pendant le travail	☐ 6 césarienne programmée	☐ 7 autre
Q13.Si césarienne, préciser l'indication principale:			

**V.COMPLICATIONS OBSTETRICALES**			*La patiente a-t-elle présenté les complications suivantes:*		
Q14.Hémorragie	☐ 1 Oui	☐ 0 Non	Q17. Infection génitale/septicémie	☐ 1 Oui	☐ 0 Non
Q15.Travail dystocique	☐ 1 Oui	☐ 0 Non	Q18.Pré-éclampsie/éclampsie	☐ 1 Oui	☐ 0 Non
Q16.Rupture utérine	☐ 1 Oui	☐ 0 Non	Q19.Autres, préciser:		
L'état de la patiente pendant son hospitalisation a-t-il nécessité :					
Q20.Une transfusion	☐ 1 Oui	☐ 0 Non	Q22.Transfert vers autre établissement	☐ 1 Oui	☐ 0 Non
Q21. Une hystérectomie	☐ 1 Oui	☐ 0 Non	Q23. Date de sortie : (jour / mois / année)		

Q24.Etat patiente après accouchement *si décédée aller à Q25 ; si vivante aller à Q27*	☐ 1 vivante	☐ 2 décédée dans cet établissement	☐ 3 décédée après transfert vers un autre établissement
Q25.Cause principale du décès:			Q26.Date du décès :

**VI.1. ETAT NOUVEAU-NÉ 1**	**VI.2. ETAT NOUVEAU-NÉ 2**	**VI.3. ETAT NOUVEAU-NÉ 3**
Q27. Etat à la naissance<br>☐ 1 vivant ☐ 2 mort-né	Q31. Etat à la naissance<br>☐ 1 vivant ☐ 2 mort-né	Q35. Etat à la naissance<br>☐ 1 vivant ☐ 2 mort-né
Q28. Poids (g): /__/__/__/__/	Q32. Poids (g): /__/__/__/__/	Q36. Poids (g): /__/__/__/__/
Q29 Sexe : Masculin / Féminin	Q33 Sexe : Masculin / Féminin	Q37 Sexe : Masculin / Féminin
Q30. Etat à la sortie<br>☐ 1 vivant ☐ 2 décédé avant 24h<br>☐ 3 décédé aprés 24h	Q34. Etat à la sortie<br>☐ 1 vivant ☐ 2 décédé avant 24h<br>☐ 3 décédé aprés 24h	Q38. Etat à la sortie<br>☐ 1 vivant ☐ 2 décédé avant 24h<br>☐ 3 décédé aprés 24h
